# A Visual Encoding Model Based on Contrastive Self-Supervised Learning for Human Brain Activity along the Ventral Visual Stream

**DOI:** 10.3390/brainsci11081004

**Published:** 2021-07-29

**Authors:** Jingwei Li, Chi Zhang, Linyuan Wang, Penghui Ding, Lulu Hu, Bin Yan, Li Tong

**Affiliations:** Henan Key Laboratory of Imaging and Intelligent Processing, PLA Strategic Support Force Information Engineering University, Zhengzhou 450001, China; shuaiyehelaowu@163.com (J.L.); zcboluo@hotmail.com (C.Z.); wanglinyuanwly@163.com (L.W.); dpenghui6@163.com (P.D.); hull852@163.com (L.H.); ybspace@hotmail.com (B.Y.)

**Keywords:** visual encoding models, deep neural networks, contrastive self-supervised learning, fMRI, visual cortex

## Abstract

Visual encoding models are important computational models for understanding how information is processed along the visual stream. Many improved visual encoding models have been developed from the perspective of the model architecture and the learning objective, but these are limited to the supervised learning method. From the view of unsupervised learning mechanisms, this paper utilized a pre-trained neural network to construct a visual encoding model based on contrastive self-supervised learning for the ventral visual stream measured by functional magnetic resonance imaging (fMRI). We first extracted features using the ResNet50 model pre-trained in contrastive self-supervised learning (ResNet50-CSL model), trained a linear regression model for each voxel, and finally calculated the prediction accuracy of different voxels. Compared with the ResNet50 model pre-trained in a supervised classification task, the ResNet50-CSL model achieved an equal or even relatively better encoding performance in multiple visual cortical areas. Moreover, the ResNet50-CSL model performs hierarchical representation of input visual stimuli, which is similar to the human visual cortex in its hierarchical information processing. Our experimental results suggest that the encoding model based on contrastive self-supervised learning is a strong computational model to compete with supervised models, and contrastive self-supervised learning proves an effective learning method to extract human brain-like representations.

## 1. Introduction

Understanding how the human brain functions is a subject that neuroscientists are constantly exploring, and the visual system is one of the most widely and deeply studied sensory systems [1]. Functional magnetic resonance imaging (fMRI) [2], which is an important non-intrusive tool for obtaining brain activity information, can reach a high spatial resolution. In addition to accurate means of physical measurement, constructing a computational model in line with brain visual information processing is an equally important element of the strategy to understand brain operation [3,4]. The visual encoding model based on fMRI is a mathematical model that simulates the process of brain visual information processing to predict fMRI activity for any visual input stimulus based on a known or assumed visual perception mechanism, and it describes the relationship between visual inputs and fMRI responses [5,6]. With the assistance of visual encoding models, the known visual assumptions can be verified, and new visual mechanisms can be explored.

In primates, visual information is processed by a cascade of neural computations [7,8]. This process is extremely complex; therefore, the mapping from the input stimulus space to the brain activity space can be regarded as nonlinear. However, due to the unclear mechanism of brain visual information processing, it is difficult to directly construct a model to characterize such nonlinear relationships; thus, a linearizing feature space is usually introduced to assist the model building [9]. The so-called linearizing feature space refers to the idea that the nonlinear mapping from the input space to the feature space contains all of the nonlinearity between the input space and the activity space, such that only one linear mapping is needed from the feature space to the activity space. Thus, the construction of the feature space is the core of the linearizing encoding model, which determines the encoding performance.

As with the early period of the computer vision domain, the traditional visual encoding models were based on a handcrafted feature space. Many works have confirmed that Gabor wavelet features effectively express the activity response of the primary visual cortex [10]. Kay et al. [11] proposed a receptive-field model based on a Gabor wavelet pyramid (GWP) with different positions, orientations, and spatial frequencies. The GWP visual encoding model is a classical low-level encoding model; however, it is not fit for the higher visual cortex. In the follow-up studies, researchers tried to employ hand-marked semantic labels to encode high-level visual areas [12,13]. The achieved encoding performance was improved compared with the GWP model but was still poor. This handcrafted fashion depends on and is limited to visual priors, and it is almost impossible to design features that fully express the processing of visual information in the brain [14].

Recently, with the rapid development of deep learning, the hierarchical information processing mechanism of a deep neural network has been shown to be highly similar to that of the visual cortex [15,16,17]; hence, the visual encoding model based on deep network features has been extensively developed [18,19,20]. In order to construct encoding models that are highly similar to human visual representations, researchers have continuously explored the feature expression of deep neural networks from the aspect of many factors, such as network structure and training tasks. Regarding the network structure, CNN has been widely proved to be effective in predicting the dorsal [21] and the ventral visual stream [22], whether for static [23,24] or dynamic visual stimuli [21,22,25,26,27]. Shi et al. [27] added recurrent connections to CNN to achieve better prediction performance for natural movie stimuli. On training tasks, object categorization is always regarded as the guiding principle for the primate ventral stream [22]; therefore, most deep feature construction is based on the classification task. Qiao et al. [28] introduced the image caption task to improve the encoding performance in high-level visual cortices. The image caption can be regarded as the task upgrading of image classification, which pays more attention to the relationships between objects instead of simply focusing on a single object. When summarizing the visual encoding models based on deep network features, we can find that the construction of the feature space has been based on supervised training methods, whereas advanced primates have more dependence on unsupervised data for learning and relatively less dependence on supervised information [29].

In biological terms, humans have about 10^14^ synapses which only live for about 10^9^ seconds. Thus, if most neurons need to be reinforced by learning, the limited external labels are unlikely to provide sufficient information. From the perspective of machine learning, on the one hand, obtaining large amounts of labeling information is difficult; on the other hand, the information contained between samples is much greater than that provided by sparse labels. Therefore, both biological intelligence and computer intelligence need unsupervised learning to help fully train neurons. As an important branch of unsupervised learning, self-supervised learning does not rely on manually annotated labels but directly utilizes supervised information provided by the example itself. In computer vision, self-supervised learning methods can be roughly divided into generative ones and contrastive ones. Inspired by the hypothesis of ‘analysis by synthesis’ [30,31], Han et al. [32] constructed the computational model of the visual cortex based on VAE [33], which is a generative self-supervised model. They argued that VAE’s encoder and decoder, respectively, simulate the bottom-up and top-down pathways in the brain; thus, VAE seemed to be a mathematical model similar to the brain visual system. However, the results of Han et al. showed that, compared with CNN, the VAE-based encoding model had relatively poor encoding performance in each visual area, especially in the advanced visual area. The greatest issue concealed behind VAE is that the model learns by measuring the pixel-level loss in the output space, thus lacking semantic learning in the training process. The most recent stage of research has seen the rapid development of contrastive self-supervised models [34,35,36,37,38,39,40,41], which have attracted wide interest by researchers. The idea behind this contrastive model can be simply understood as a similarity comparison which learns the feature representation that renders elements similar or different. The contrastive self-supervised model approaches the supervised model in performance in downstream image classification tasks, showing its powerful ability to obtain information from data. Zhuang et al. [42] constructed different types of unsupervised models to predict macaques’ neural response and showed that contrastive embedding methods achieve equal or even better prediction performance than supervised methods. This indicates that contrastive self-supervised learning is a strong candidate for the simulation of learning mechanisms in the brain.

Inspired by the unsupervised visual mechanism in the human brain, this paper constructs a contrastive self-supervised visual encoding model based on fMRI utilizing pre-trained neural networks as fixed feature extractors. We discuss and analyze the encoding performance of encoding models based on contrastive self-supervised learning and supervised classification tasks from the perspective of the learning objective and learning rule. Our research can also promote the understanding of the contrastive self-supervised model while taking the visual encoding perspective.

## 2. Materials and Methods

### 2.1. Experimental Data

The experimental data used in this article were sourced from a previously published study [43] and are available at the Kamitani Laboratory website: https://github.com/KamitaniLab/GenericObjectDecoding (20 July 2021). The experiment collected five subjects’ brain fMRI activity while they were viewing natural color images, which were obtained from the online database ImageNet [44]. The training set contained 1200 images from 150 representative categories (eight images per category), while the validation set included 50 images from 50 different categories (one image per category). The fMRI data were divided into seven distinct visual regions of interest, including V1, V2, V3, V4, the lateral occipital complex (LOC), the parahippocampal place area (PPA), and the fusiform face area (FFA). The V1–V4 regions were divided by retinotopy experiments, and the V1–V3 regions were defined as the lower visual cortex (LVC). Meanwhile, the LOC, PPA, and FFA regions were defined as the higher visual cortex (HVC), which were determined by the functional localizer experiments. More details about the fMRI dataset on ImageNet can be found in a previous paper [43]. In this article, the preprocessed data were used for analysis.

### 2.2. Overview of the Proposed Method

**Figure 1:**
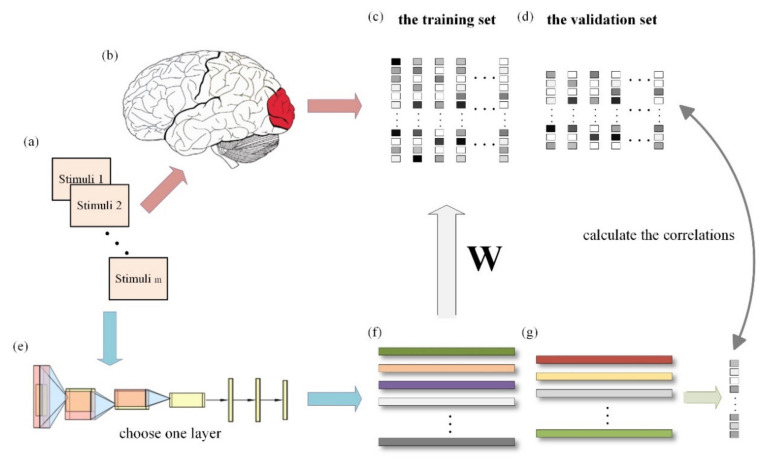
General process of visual encoding based on deep network features. (**a**) External visual stimuli. (**b**) Information processing of visual cortex. (**c**) fMRI responses of the training set data. (**d**) fMRI responses of the validation set data. (**e**) The pre-trained deep neural network. (**f**) Features extracted from training set samples. (**g**) Features extracted from validation set samples. When the external visual stimuli are processed by the brain visual cortex, the activity responses can be measured by fMRI. The features of the stimuli can be extracted from the middle layers of the pre-trained deep network. A linear regression model is trained for each voxel by using the fMRI responses and the extracted features of the training set data. After training, we can obtain a weight matrix W for each voxel. Subsequently, the predicted fMRI responses are obtained with the extracted features of the validation set data and the pre-trained W, and the correlations are calculated with the real responses.

In this paper, we used the linearizing visual encoding approach to construct a visual encoding model based on contrastive self-supervised learning and a visual encoding model based on supervised classification tasks. The specific methods are outlined below, and Figure 1 presents the overall process. Firstly, the features of all sample images (including the training set and validation set) were extracted with the pre-trained deep network model. Secondly, using the feature–response pairs in the training set, a linear regression model from features to voxel responses was trained for each voxel in each ROI (i.e., V1, V2, V3, V4, LOC, PPA, and FFA). Finally, the linear model trained in the previous step was used to predict the voxel response of each voxel within each region against the validation set, and the correlations were calculated with the true voxel responses. The prediction ability of different visual encoding models for distinct visual regions can be displayed by comparing the correlations. Each step will be introduced in detail in the following sections.

### 2.3. Extracting Hierarchical Features from Pre-Trained Models

Contrastive self-supervised learning generally includes four important modules: (1) data augmentation module; (2) encoder module; (3) representation extraction module; and (4) loss function module. The anchor points, positive examples, and negative examples can be created through data augmentation (flip, rotation, color distortion, etc.). Meanwhile, data augmentation can help to extract the invariant features in the input samples. Encoders are specially designed neural networks (such as a residual network, ResNet) to extract hierarchical feature representations from input images. Representation is the unique property of an object, which allows the model to understand the commonality of similar samples, as well as the heterogeneity of dissimilar samples. The contrastive self-supervised model can be trained by using the representations extracted from the samples to construct a suitable loss function and perform backpropagation. This paper used the SimCLRv2 (an improved version of SimCLR [41], a simple framework for contrastive learning of visual representations) model proposed by Google Research’s Brain Team (2020) [45] to construct a visual encoding model based on contrastive self-supervised learning. The SimCLRv2 learns general representations by maximizing the consistency between different views of the same sample and the distance between negative examples and anchor points. In this model, the data augmentation module is a combination of random crop (with resize and random flip), random color distortion, and random Gaussian blur. The encoder in the network is based on the ResNet architecture (up to the final average pooling layer) with different depths and widths. In each training batch, the representations of positive and negative samples are extracted by adding a learnable nonlinear transformation network after the encoder, and the NT-Xent loss function (see details in [41]) is used to calculate the loss. When one training batch is completed, the nonlinear transformation network is discarded, and the same untrained network is added in the next training batch. The model also uses the memory mechanism to increase the number of contrasting negative examples.

In this study, we used the encoder network ResNet50 [46] of the SimCLRv2 model to extract the features of the sample image. The deep convolutional neural network can extract low-level, intermediate-level, and high-level features of the image. The more layers of the network, the richer the feature levels that can be extracted. A deeper network extracts more abstract features, which contain more semantic information. Compared with AlexNet [47] and VGG16 [48], ResNet50 uses the shortcut connection to introduce residual units to construct a deeper network structure; thus, it can extract richer feature hierarchies. It contains a convolution layer, four convolution modules (including 3, 4, 6, and 3 bottlenecks), and a fully connected classification layer. We extracted features from 18 different layers for each input sample, including the outputs of the first convolution layer, 16 bottlenecks, and the final average pooling layer. Each layer of the feature was used to construct the encoding model. The performance of different layers determines their ‘matching performance’ with different visual region voxels, and the feature layer best suited for prediction is defined as the optimal feature layer. In order to carry out a fair comparison with the self-supervised model, we used the supervised classification model trained by the same team in the same ResNet50 structure. For the sake of convenience, we denoted the ResNet50 trained in a contrastive self-supervised learning manner as ResNet50-CSL. Meanwhile, in SimCLRv2, the researchers also fine-tuned the whole base network on 1%, 10%, and 100% of labeled examples from ImageNet. The internal representations learned by the five models (ResNet50, ResNet50-CSL, Fine-tuned-1%, Fine-tuned-10%, and Fine-tuned-100%) were compared to the ventral visual representation in the human brain. All of the pre-trained models can be downloaded from https://github.com/google-research/simclr (20 July 2021).

### 2.4. Voxel-Wise Linear Regression Mapping

A linear regression model was constructed for a single voxel, and the model can be expressed by the formula

(1) y=Xw+ε,

where *y* represents single voxel responses, *X* represents the features extracted from the pre-trained models, *w* represents the weights obtained from model training, and *ε* represents the noise term. Here, *y* is an *m* × 1 matrix, where 𝑚 denotes the number of samples; *X* is an *m* × (*n* + 1) matrix, where 𝑛 denotes the number of features, and the last column serves as a constant term; and 𝑤 is an (*n* + 1) × 1 matrix. The number of features *n* is much larger than the number of samples *m*, and when *n* is particularly large, it contains more noise; therefore, we consider that most features do not contribute to the model, and it is beneficial to use a small part of the features via regularization. We first performed dimensionality reduction by PCA to compress the image feature into 1249 dimensions and then used the sparse linear regression model to fit the data. The regularized orthogonal matching pursuit (ROMP) algorithm [49] was finally selected by comparing the commonly used linear regression models. When the sparse linear regression model training was completed, the parameter weight 𝑤 could be used to predict the voxel response of the validation set. The completion of this step marked the end of the visual encoding model building process.

### 2.5. Quantitative Analysis of Models

For each voxel, the prediction accuracy was used as an index for evaluating the predictive performance, and its formula is as follows:

(2) $$\rho =cor\left(r,\hat{r}\right),$$

where *ρ* represents the Pearson correlation coefficient between the real response *r* and the predicted response $\hat{r}$ for a single voxel across all 50 images in the validation set. We randomly scrambled the correspondence between the measured and predicted voxel responses and then recalculated the prediction accuracy. After repeating this process 1000 times, we finally defined the voxels with a prediction accuracy higher than 0.41 (*p* < 0.001, randomization test) as voxels that can be accurately predicted. In order to comprehensively compare the advantages of different models (percent of voxels with higher prediction accuracy) for a certain visual region, we randomly (with a 0.5 probability) permuted the prediction accuracy of voxels in one ROI that can be accurately predicted by both models and then recalculated the advantage. We repeated this process 1000 times to generate a null hypothesis distribution. When the advantage of one model was over *x* (*p* < 0.05, permutation test), it was considered that this model was significantly better than the other, where *x* was determined by the number of voxels. We also calculated the noise ceiling by Monte Carlo simulations, as described in [50]. The signal and the noise were assumed to follow a normal distribution, and the two were independent. The noise was zero mean, and its variance was assumed to be the pooled squared standard error of 35 repeated test runs. We calculated the mean of the measured fMRI responses to be the mean of the signal. The variance of the signal was obtained by subtracting the variance of the noise from the variance of the measured fMRI responses. We performed 1000 simulations on the basis of a known distribution and calculated the prediction accuracy. Finally, the median value was taken as the noise ceiling.

### 2.6. Representational Similarity Analysis

Considering that linear regressions on individual voxels tend to be noisy, we also showed the representational similarities between model representations and brain representations to evaluate the model’s ability of characterizing brain representations. A representational dissimilarity matrix (RDM) is commonly used to describe the model or brain representations, which is computed by the correlation distance (1—Pearson correlation) between all pairs of stimuli representations. In each brain ROI, we calculated a 50 × 50 representational dissimilarity matrix for each subject based on the 50 testing stimuli. Accordingly, we computed layer-wise RDMs for each layer in each model. Kendall’s rank correlation was used to judge the similarity between a model RDM and a brain RDM. We also defined the inter-subject brain RDM correlation as the average Kendall rank correlation between the 5 subjects for comparison with model-to-brain RDM correlations.

## 3. Results

**Figure 2:**
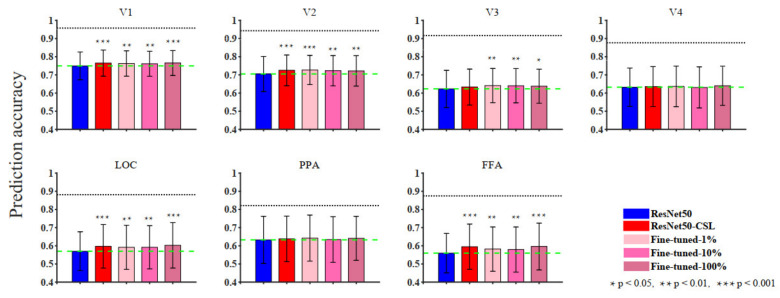
The ROI-level encoding performance of supervised, self-supervised, and semi-supervised models. The prediction accuracy for seven ROIs of each model is summarized in the histogram. Bars show the average prediction accuracy of 500 voxels (top 100 voxels from each subject) in each ROI. Different colors represent different models. The black horizontal lines represent the median noise ceiling, and the green dashed lines indicate the median values of the ResNet50 encoding model. The statistical significances (two-sample *t*-test) are marked on the figure to show whether the other models perform significantly better than the ResNet50 encoding model.

**Figure 3:**
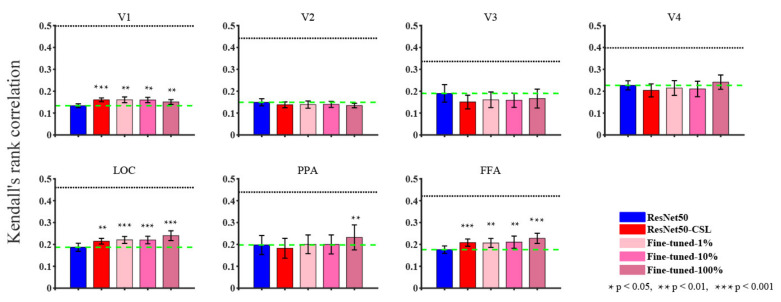
The ROI-level representational performance of supervised, self-supervised, and semi-supervised models. The bar diagrams with different colors are based on the mean and variance of model-to-brain RDM correlations of the most predictive feature layer across five subjects for each ROI. The black dotted line in each panel indicates the inter-subject brain RDM correlation for each brain area, and the green dashed line represents the mean correlation of the ResNet50 model. The statistical significances (paired *t*-test) are marked on the figure to show whether the other models perform significantly better than the ResNet50 model.

**Figure 4:**
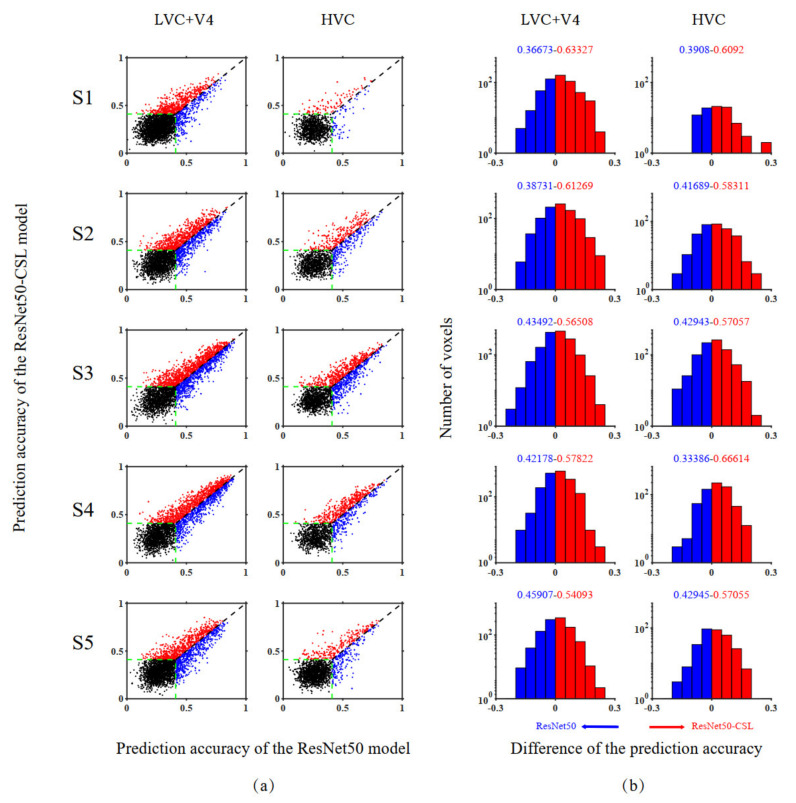
Comparisons of encoding performance between ResNet50-CSL and ResNet50 for five subjects. (**a**) Voxel-wise comparisons of the two models in prediction accuracy. The two axes of each row display a comparison between the prediction accuracy of the two models for one subject. The ordinate and abscissa values of each point in the scatter plot represent the prediction accuracy of ResNet50-CSL and ResNet50, respectively. The red dots correspond to voxels that can be predicted more accurately by ResNet50-CSL than ResNet50, and vice versa for the blue dots. The black dots represent voxels that cannot be accurately predicted by either model, and the green dashed lines indicate the accuracy threshold (0.41, *p* < 0.001, randomization test). (**b**) Distribution of prediction accuracy difference between ResNet50-CSL and ResNet50. The red bars above 0 indicate the distribution of voxels with higher prediction accuracy for ResNet50-CSL, and vice versa for the blue bars of ResNet50. It should be noted that the voxels in the distribution plot are those that can be accurately predicted by both models. The colored number on each side represents the fraction of voxels whose prediction accuracy is higher under that model.

**Figure 5:**
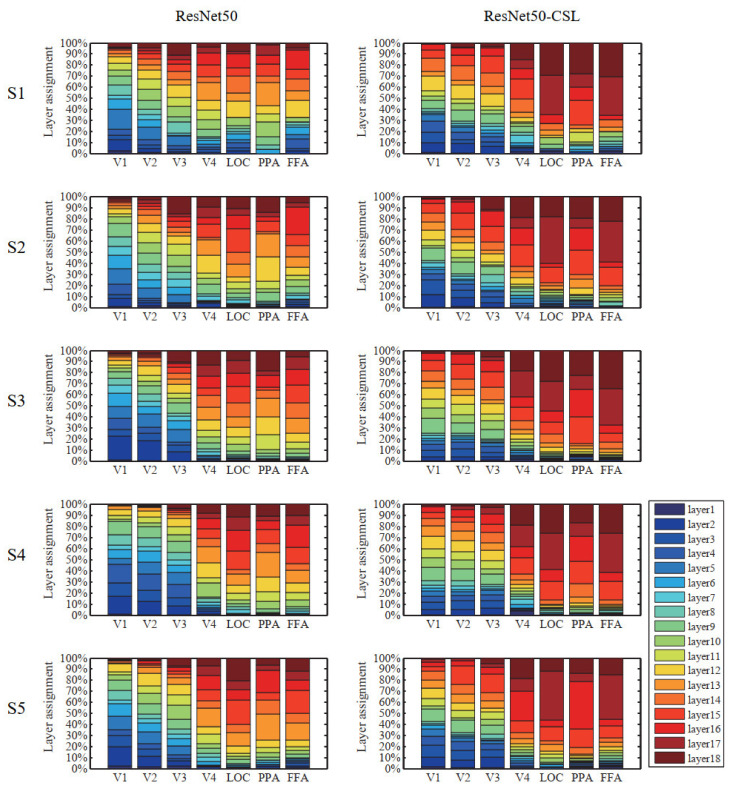
Distribution of optimal feature layers in each ROI across five subjects. The colored bars within each column indicate the contributions of feature layers of different models to predicting a single ROI. The different colors represent different layers. The left side in the figure represents ResNet50, and the right side represents ResNet50-CSL. From LVC to HVC, the low-level features account for an increasingly smaller fraction, and the high-level features account for an increasingly larger fraction of the total regardless of the model.

In Section 3, we start by showing the ability of five models to encode (Figure 2) and represent (Figure 3) the brain in terms of prediction accuracy and RDM correlation. We plot the histogram to compare the average prediction accuracy and the average Kendall rank correlation. Then, we further analyze the encoding performance between ResNet50 and ResNet50-CSL from two perspectives. Firstly, a scatter plot (Figure 4a) is used to clearly display the prediction accuracy of each voxel, and a distribution plot (Figure 4b) is used for showing the encoding difference between the models. Secondly, the percentage of each feature layer in different ROIs (Figure 5) is plotted to show their encoding contributions.

### 3.1. Overall Comparisons in Encoding and Representational Performance of Different Visual Areas between Models

In order to comprehensively analyze the encoding performance of supervised, self-supervised, and semi-supervised models, we compared the prediction accuracy between different models along the ventral visual hierarchy. Since each ROI contained many noise voxels, we selected the top 100 voxels with the highest prediction accuracy in each visual ROI across five subjects and calculated their mean and variance. As shown in Figure 2, the blue pillars represent the ResNet50 model, the red pillars represent the ResNet50-CSL model, the pink pillars represent the Fine-tuned-1% model, the hot pink pillars represent the Fine-tuned-10% model, and the violet-red pillars represent the Fine-tuned-100% model. Overall, the gap in encoding performance between the five models was not large, and the ResNet50 encoding model performed slightly worse than the self-supervised and fine-tuned models in multiple visual cortical areas (V1, V2, LOC, and FFA).

Figure 3 presents model-to-brain RDM correlations without linear mixing of the features, and the reported values are the average of the maximum correlations across five subjects. As it can be seen from Figure 3, the self-supervised and semi-supervised models yielded more human brain-like representations in V1, LOC, and FFA. From both perspectives of RDM correlations and prediction accuracy, the self-supervised and semi-supervised models had distinct advantages in areas V1, LOC, and FFA, and the Fine-tuned-100% model exhibited a relatively better performance.

### 3.2. Voxel-to-Voxel Comparisons of Prediction Accuracy between the ResNet50-CSL and the ResNet50 Encoding Models

In order to compare the encoding performance between self-supervised and supervised encoding models in a voxel-to-voxel manner, a scatter plot was prepared showing the prediction accuracy of different models for a single voxel, as well as a distribution plot showing the difference in prediction performance between the models. Figure 4 presents the model comparison results across five subjects. The exact number of advantages of different models and their significant indicators are detailed in Table 1. It can be concluded that the ResNet50-CSL encoding model shows an obvious advantage (more than *x*, *p* < 0.05, permutation test) in all subjects for LVC (V1, V2, V3), V4, and HVC (LOC, PPA, FFA), which reflects its robustness. In the scatter plot (see Figure 4a), the number of red dots that represent the more accurately predicted voxels by ResNet50-CSL was more than the number of blue dots that represent the more accurately predicted voxels by ResNet50, and the accuracy threshold here is 0.41. Accordingly, in the distribution plot (see Figure 4b), the occupancy rate of the red bars that represent ResNet50-CSL is about 15–30% higher than that of the blue bars that represent ResNet50. Therefore, compared with the supervised encoding model, the contrastive self-supervised encoding model can effectively improve the encoding performance of both LVC and HVC.

### 3.3. Contributions of Different Feature Layers to Encoding Performance

**Figure 6:**
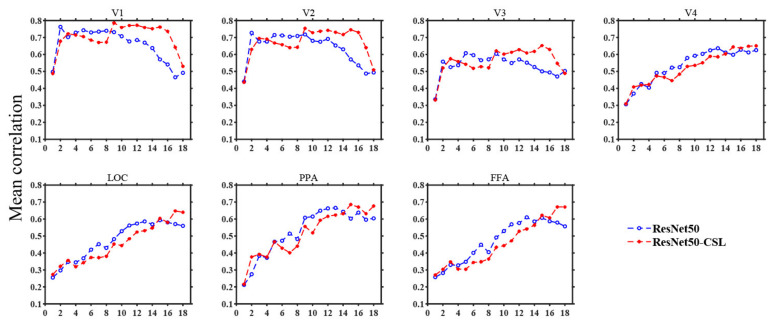
Average prediction accuracy changes in 18 feature layers in each ROI (Subject 3). In each subplot, the abscissa denotes different layers of deep neural networks, and the ordinate denotes the mean prediction accuracy of the top 100 voxels with the highest correlation. The ResNet50 model is represented by blue broken lines, and the ResNet50-CSL model is represented by red broken lines. As the layers become deeper, the prediction accuracy of LVC first rises and declines afterward, and it rises for HVC.

During the construction of the encoding model, an optimal feature layer was selected for each voxel from 18 middle layers of ResNet50 or ResNet50-CSL. For the voxels that can be accurately predicted in each region, we plotted the distribution of the optimal feature layers in each ROI across five subjects. Meanwhile, in order to show the encoding ability of different feature layers more clearly, the average prediction accuracy changes in 18 feature layers in each ROI were plotted. As indicated in Figure 5, regardless of the encoding model, it was distinctive that LVC used more low-level features, and HVC used more high-level features. This property is indicated in the broken line change diagram, as detailed below. The average prediction accuracy of the two models shows a trend of first increasing and then decreasing in LVC, while it shows an increasing trend in HVC. This indicates that ResNet50 is similar to the human visual cortex in its hierarchical structure, regardless of whether the supervised or the self-supervised training method was selected. Moreover, it can be established that the ResNet50-CSL model made more use of the intermediate- to high-level feature layers than the ResNet50 model for each ROI. In LVC, ResNet50 mainly used the feature layers below layer12 to encode, while for ResNet50-CSL, the feature layers above layer12 also accounted for a large proportion. Accordingly, as seen in Figure 6, the feature layers with a high average prediction accuracy of ResNet50-CSL were distributed in layer10–layer16, while for ResNet50, they were distributed in layer2–layer10. Compared with ResNet50, ResNet50-CSL made more use of the last four layers for HVC, and the last two layers for LOC and FFA. Correspondingly, we can see that the average prediction accuracy of the last four layers of ResNet50-CSL was significantly higher than that of the other layers.

## 4. Discussion

### 4.1. Contrastive Self-Supervised Model vs. Supervised Classification Model

Consistent with our previous indicated results, the ResNet50-CSL encoding model achieved an equal or even relatively better encoding performance than the ResNet50 encoding model in multiple visual cortical areas, suggesting that the contrastive self-supervised model is a strong contender for explaining ventral stream visual representations. In the deep neural network, the model architecture, the learning objective, and the learning rule all affect the feature expression of the model [51], and the feature expression is central to visual encoding. Next, we will analyze the possible causes of the encoding differences between the contrastive self-supervised model and the supervised classification model from these three aspects.

The model architecture has no special differences; both models used the ResNet50 architecture (up to the final average pooling layer) as the feature extractor. The only difference is that the ResNet50-CSL model employed additional projection headers during training to extract representations for calculating the loss, while the supervised ResNet50 model added a fully connected layer at the end of the network to perform classification tasks. From the perspective of the training objective, the ResNet50-CSL model was trained in a task-agnostic way, while the ResNet50 model took the classification task as the goal driven to extract the invariance. Visual categorization is always regarded as the guiding principle for the primate ventral stream, while contrastive embedding objectives leverage augmented images to create image embeddings respecting differences at a finer scale than possible with only object categories. In our results, the contrastive self-supervised models can perform as well or even better than the supervised models. This indicates that brain-like representations may need to require the ability of distinguishing between different views which are not limited to the category level. In terms of the learning rule, the greatest difference between the two models is the method of either self-supervised or supervised training. Supervised learning uses manual labels to provide guidance for training. This learning method is often affected by labels, and only part of the image is concerned. The label of the sample is subjectively affected at the same time. Self-supervised learning makes the model discover information and extract useful features from the samples by itself. Theoretically, this learning method can learn more abundant and objective information. Our encoding results also prove that contrastive self-supervised learning is an effective learning method to extract reliable information from samples. In fact, the contrast mechanism is an important learning mechanism of the human brain. When we observe an object, we do not know what the item is in isolation, but we still understand the connections and differences between such objects. For example, we can easily distinguish between an apple and a banana, even if we do not know what an apple and a banana are.

### 4.2. Exploring Feature Layers of ResNet50-CSL from Visual Encoding

The results of Section 3.3 show that layer10–layer16 of ResNet50-CSL had a high average prediction accuracy in LVC, indicating that the intermediate feature layers of ResNet50-CSL contained rich low-level visual features. Meanwhile, for HVC, ResNet50-CSL made more use of the last four layers, and the average prediction accuracy of these layers was significantly higher than that of the other layers. This indicates that the last few layers of ResNet50-CSL were more suitable for explaining the visual representation of the higher ventral stream than the other layers, meaning that most voxels in HVC could be optimally encoded by relying only on the last few layers. However, the ResNet50 model could not simply rely on the last features, and many optimal feature layers came from the intermediate layers, indicating that its last features could only meet the needs of part of the voxels in HVC.

Further analysis was carried out from the perspective of the training target. The supervised ResNet50 model took the classification task as the target for training, and the features trained by CNN from the low level to the high level increasingly served this target; therefore, the features extracted at higher layers are more conducive to performing classification tasks. The visual system in the human brain, however, is highly developed, can perform multiple visual tasks, and is not limited to a single classification task. In comparison with the visual system in the human brain, the high-level features of the supervised classification model lost too much information to perform other tasks, and thus only some voxels in HVC could be optimally encoded. In contrast, the contrastive self-supervised model does not perform a specific task but likely learns rich feature representations through contrast mechanisms for downstream tasks. Therefore, the high-level features of the self-supervised model can perform multiple tasks rather than a single task, which is more similar to the visual information processing method of the human brain; this may also explain why its last few layers could optimally encode more voxels in HVC.

### 4.3. Future Development Directions

The visual encoding model constructed in this paper has good encoding performance for LOC and FFA but shows no significant advantage for PPA. We believe that data augmentation is the key to prediction accuracy. It is likely that the data augmentation used by SimCLR is not suitable for the visual representation extraction of PPA; hence, the encoding performance of PPA was not significantly improved. Data augmentation is an important module of contrastive self-supervised learning, which can extract invariant features in the representation space. In further studies, different encoding models can be constructed using different data augmentation methods, and the expression of invariant features can be explored in different ROIs. In addition, the brain visual information processing does not solely rely on a certain learning method; a purely supervised or unsupervised learning method is unlikely to occur in primates. From the results of Section 3.1, the Fine-tuned-100% model exhibited a relatively better performance in most visual areas than the self-supervised and supervised models. Therefore, in future studies, it is necessary to combine supervised and unsupervised training methods to construct brain-inspired computing models.

## Figures and Tables

**Table 1:** Comparisons of advantages between different encoding models across five subjects.

Subject	Visual Cortex	Advantage of ResNet50	Advantage of ResNet50-CSL	Significance Indicators
S1	LVC + V4	36.67%	**63.33%**	53.49%
	HVC	39.08%	**60.92%**	58.62%
S2	LVC + V4	38.73%	**61.27%**	52.74%
	HVC	41.69%	**58.31%**	54.50%
S3	LVC + V4	43.49%	**56.51%**	52.06%
	HVC	42.94%	**57.06%**	52.87%
S4	LVC + V4	42.18%	**57.82%**	52.19%
	HVC	33.39%	**66.61%**	53.32%
S5	LVC + V4	45.91%	**54.09%**	52.77%
	HVC	42.95%	**57.05%**	54.60%

The results of the model with significant advantages are in bold.

## Data Availability

The detailed information about the fMRI data is provided in previous studies, and the public dataset can be downloaded from https://github.com/KamitaniLab/GenericObjectDecoding (20 July 2021).
